# Pharmacological strategies to prevent haemodynamic changes after intubation in parturient women with hypertensive disorders of pregnancy: A network meta-analysis

**DOI:** 10.7150/ijms.54002

**Published:** 2021-01-01

**Authors:** Sang Won Yoon, Geun Joo Choi, Hee-Kyeong Seong, Myeong Jong Lee, Hyun Kang

**Affiliations:** 1 Department of Anaesthesiology and Pain Medicine, Chung-Ang University College of Medicine, Seoul, Republic of Korea; 2 Department of Anaesthesiology and Pain Medicine, Konkuk University Medical School, Chungju, Republic of Korea

**Keywords:** Caesarean section, pregnancy-induced hypertension, intubation, pregnancy

## Abstract

***Objective***: This network meta-analysis (NMA) aimed to determine the relative efficacy and safety of pharmacological strategies used to mitigate haemodynamic instability by intubation for general anaesthesia in hypertensive parturient women undergoing caesarean section.

***Methods***: We considered randomised controlled studies comparing the effects of pharmacological strategies used to alleviate haemodynamic instability during intubation in parturient women with hypertensive disorders of pregnancy. The primary endpoints were maximum blood pressure and heart rate after intubation, and secondary endpoints were the Apgar scores at 1 and 5 min. NMA allowed us to combine direct and indirect comparisons between strategies.

***Results***: Twelve studies evaluating nine pharmacological strategies in 619 patients were included. According to the surface under the cumulative ranking curve, the maximal mean arterial pressure was lowest for high-dose remifentanil (99.4%) followed by nitroglycerin (73.6%) and labetalol (60.9%). The maximal heart rate was lowest for labetalol (99.9%) followed by high dose of remifentanil (81.2%) and fentanyl (61.6%). Apgar score at 1 min was higher with low-dose than with high-dose remifentanil (mean difference, 0.726; 95% confidence interval, 0.056 to 1.396; I^2^=0.0%).

***Conclusions***: High-dose remifentanil produces minimum blood pressure changes, while labetalol is most effective in maintaining normal heart rate in parturient women with hypertensive disorders of pregnancy during caesarean section under general anaesthesia.

## Introduction

Intubation during the induction period in general anaesthesia activates the sympathetic nervous system, resulting in haemodynamic instability, including hypertension and tachycardia 1. These reflex haemodynamic responses are exaggerated in patients with hypertension history and constitute major concerns for anaesthesiologists 2-4. For patients with hypertensive disorders of pregnancy, such as preeclampsia and gestational hypertension, intubation is often circumvented during caesarean section by performing local anaesthesia. However, due to certain conditions present in preeclampsia, including coagulopathy and thrombocytopenia, general anaesthesia is unavoidable in some patients**.** These patients exhibit a marked increase in blood pressure and heart rate during intubation and airway manipulation, which may result in elevated intracranial pressure, cerebral haemorrhage, cardiac failure, and pulmonary oedema 5. These events increase the risk of morbidity and mortality for both the mother and child.

Several types of medication have been used by anaesthesiologists to mitigate the haemodynamic reflex after airway manipulation in parturient women with hypertensive disorders of pregnancy. Opioids such as remifentanil, fentanyl, and alfentanil, and antihypertensive drugs such as nitroglycerin, labetalol, and hydralazine have been prescribed, and their effectiveness and efficacy in controlling blood pressure and heart rate with minimal effects on foetuses have been compared. Numerous studies have investigated the most effective drugs that ensure haemodynamic stability without compromising the foetus in patients with preeclampsia. However, each study was limited to the comparison of two or three drugs, and the results were inconsistent.

Therefore, we reviewed all articles that compared the effects of different drugs used to alleviate haemodynamic instability during intubation in parturient women with hypertensive disorder of pregnancy, and conducted a network meta-analysis (NMA). NMA supplements traditional meta-analysis by combining both direct and indirect comparisons between pharmacological interventions, and generates an intervention ranking for each endpoint. Our primary endpoints were the maximum changes in blood pressure and heart rate after intubation, and the secondary endpoints were the Apgar scores of newborns at 1 and 5 min.

## Methods

We developed the protocol for this systematic review and NMA according to the preferred reporting requirements for systematic review and meta-analysis protocol (PRISMA-P) statement 6. We registered the protocol in the PROSPERO network (registration number: CRD42019136067; www.crd.york.ac.uk/prospero) on 02 June 2019, and published it in a peer-reviewed journal 7. This study was performed according to the protocol recommended by the Cochrane Collaboration 8, and reported according to the PRISMA extension for NMA guidelines 9.

### Eligibility Criteria

We included RCTs comparing two or more pharmacological strategies. The PICO-SD information was as follows:

(1) Patients (P): Parturient women with hypertensive disorders of pregnancy undergoing caesarean section under general anaesthesia; those undergoing surgery under regional anaesthesia were excluded.

(2) Intervention (I): Pharmacological strategies to prevent haemodynamic changes after intubation for general anaesthesia

(3) Comparison (C): other pharmacological strategies, placebo, or no treatment

(4) Outcome measurements (O):

(1) Effectiveness

Primary endpoints were maximal mean arterial pressure (MMAP) and maximal heart rate (MHR) after intubation. Maximal systolic arterial pressure (MSAP) and diastolic arterial pressure (MDAP) were also assessed. MMAP, MHR, MSAP and MDAP after intubation were considered as the maximum value of the endpoints, not maximum change of them.

(2) Safety

Apgar scores at 1 and 5 min after delivery were assessed.

(5) Study design (SD): We included peer-reviewed RCTs without language or date limitations. Review articles, case reports, case series, letters to the editor, commentaries, proceedings, laboratory science studies, and other non-relevant studies were excluded.

### Information Sources

We searched MEDLINE, Embase, the Cochrane Central Register of Controlled Trials (CENTRAL), and Google Scholar, from their launch to November 2019, using search terms such as, and related to, 'pregnancy induced hypertension', 'caesarean section' and 'haemodynamic change'. The search strategy, which included a combination of free text, Medical Subject Heading (MeSH) terms, and EMTREE terms, is outlined in the Supplemental Digital Content. Two authors (SWY and GJC) screened the titles and abstracts of the retrieved articles. Reference lists were imported to Endnote software 8.1 (Thompson Reuters, CA, USA) and duplicate articles were removed. Additional relevant articles were identified by scanning the reference lists of articles obtained from the original search.

### Study Selection

Titles and abstracts were reviewed independently by two investigators. To minimise data duplication due to multiple reporting, papers from the same author, organisation, or country were compared. For articles determined to be eligible based on the title or abstract, the full paper was retrieved. All abstracts not providing sufficient information regarding the eligibility criteria were selected for full-text evaluation. Potentially relevant studies chosen by at least one investigator were retrieved and the full text was evaluated. Articles meeting the inclusion criteria were assessed separately by two authors (SWY and GJC), and disagreements were resolved through discussion or with the help of a third investigator (HK).

The degree of agreement between the two authors was calculated using the kappa statistics. Kappa values were interpreted as follows: less than 0: no agreement; 0.01-0.20: slight agreement; 0.21-0.40: fair agreement; 0.41-0.60: moderate agreement; 0.61-0.80: substantial agreement; and 0.81-1.00: almost perfect agreement 10.

### Data Extraction

Using a standardised extraction form, the following data were extracted independently by two investigators (SWY and GJC) and then cross-checked: (1) title; (2) author names; (3) journal name; (4) publication year; (5) study design; (6) clinical trial registration; (7) competing interests; (8) country; (9) risk of bias; (10) number of patients; (11) drugs and doses compared; (12) age of parturient women; (13) weight of parturient women; (14) height of parturient women; (15) duration of anaesthesia; (16) American Society of Anaesthesiologists' physical status score; (17) inclusion criteria; (18) exclusion criteria; (19) drugs used for induction; (20) MSAP; (21) MMAP; (22) MDAP; (23) MHR; and (24) Apgar scores. Disagreements were resolved with the aid of a third investigator (HK).

Data were extracted from the tables or text. Missing information was calculated from the available data, or extracted from the figures using the open source software Plot Digitizer (version 2.6.8; http://plotdigitizer.sourceforge.net). Because the studies comparing the effect of remifentanil used different doses, remifentanil groups were categorised into low-dose (< 1 mcg/kg) and high-dose (≥ 1 mcg/kg).

### Study Quality Assessment

The quality of the studies was independently assessed by two authors (MJL and HK) using the Revised Cochrane risk of bias tool for randomised trials (RoB 2.0) 11. The risk of bias (ROB) was evaluated by considering the following domains: (1) bias arising from the randomisation process; (2) bias owing to departures from the intended interventions; (3) bias from missing outcome data; (4) bias in measurement of the outcome; and (5) bias in selection of the reported results, including deviations from the registered protocol. The response options for each risk of bias judgement were 'low risk of bias', 'some concerns', and 'high risk of bias'.

The overall ROB was evaluated according to these domain-level judgements 11. We rated trials at: (1) low risk of bias overall if all domains were rated as low risk of bias; (2) some concerns overall if at least one domain was rated as some concerns; and (3) high risk of bias overall if at least one domain was rated as high risk of bias or some concerns for multiple domains in a way that substantially lowered confidence in the result.

### Statistical Analysis

Ad-hoc tables were designed to summarise data from the studies and show their key characteristics and important questions related to the review objectives. After extracting the data, we determined the feasibility of a meta-analysis. Specifically, we evaluated the heterogeneity and transitivity assumptions by examining the comparability of eligibility criteria and patient demographics and the ROB as potential treatment-effect modifiers across comparisons 12.

When the treatment nodes formed a connected network of evidence, we performed NMA. A multiple treatment comparison NMA, which is a generalisation of methods used in meta-analysis, includes both direct and indirect comparisons between treatments. An NMA based on a frequentist framework was performed using the NMA graphical tools by Chaimani et al. 13. Given the heterogeneity of populations and methods among the included trials, we used the random-effects model in our primary analysis.

A network plot linking all included pharmacological strategies was created to indicate the type of pharmacological strategy, number of patients under different pharmacological strategies, and number of pair-wise comparisons. The nodes of the network plot indicate the pharmacological strategies being compared, while the edges indicate the available direct comparisons between pharmacological strategies. Each drug or drug combination was treated as a node in this network. Nodes and edges were weighted on the basis of the number of parturient women and the inverse of the standard error of the effect.

Contribution plots were used to represent the percent contribution of each estimate in the summary estimate and the entire network. We displayed the contribution percentage of each comparison by weighted squares in the contribution plots.

We examined the consistency of the total network through global and local tests of inconsistency. We evaluated the global consistency assumption using the design-by-treatment interaction model 14. We also evaluated each closed loop in the network to examine local inconsistencies between direct and indirect effect estimates for the same comparison. In each loop, we estimated the inconsistency factor (IF) as the absolute difference (with 95% confidence interval [CI] and a z-test) between direct and indirect estimates for each paired comparison. The IF is the logarithm of the ratio of two odds ratios (RoR) from the direct and indirect evidence in the loop; RoR values close to 1 indicate agreement.

We also showed the relative treatment effects between all active pharmacological strategies using ranked forest plots. The mean summary effects with CIs are presented together with their predictive intervals to facilitate the interpretation of the results in light of the magnitude of heterogeneity. Predictive intervals provide an interval expected to encompass the estimate of a future study.

Rankograms and cumulative ranking curves were drawn for each pharmacological strategy. A rankogram plots the probabilities for treatments to assume any possible rank among all treatments evaluated in the NMA. We used the surface under the cumulative ranking curve (SUCRA) value to present the hierarchy of pharmacological strategies. The SUCRA is a relative ranking measurement that accounts for the uncertainty in the treatment order, by taking into account both the location and variance of all relative treatment effects 15. The SUCRA can assume values up to 100%, with higher values suggesting better pharmacological strategies.

We tested for small study effects and publication bias using the comparison-adjusted funnel plot 16. As the number of included studies was fewer than 10 for all outcomes except MHR, publication bias was not assessed for these outcomes.

If only two groups were compared for certain outcomes, a pair-wise meta-analysis was conducted to generate summary estimates and assess statistical heterogeneity across the included studies. Summary estimates were reported as mean differences (MDs), standardised mean differences, or relative risks, as appropriate, with the corresponding 95% CIs. Heterogeneity between studies was assessed using the Cochran's Q and the Higgins I^2^ statistics. A level of 10% significance (P < 0.10) in the chi‑squared statistics or an I^2^ greater than 50% indicated considerable heterogeneity, and the corresponding data were analysed using the Mantel-Haenszel random-effects model; otherwise, the Mantel-Haenszel fixed-effects model was applied 17. All statistical analyses were performed using Stata SE, version 15.0 (StataCorp, College Station, TX).

### Evidence Synthesis

The evidence grade was determined using the guidelines of the Grading of Recommendations, Assessment, Development, and Evaluation (GRADE) system, which uses a sequential assessment of evidence quality followed by an assessment of the risk-benefit balance and a subsequent judgement on the strength of the recommendation 18. Two authors (MJL and HK) with experience in using GRADE rated each domain for each comparison separately and resolved discrepancies by consensus. We rated the certainty for each comparison and outcome as high, moderate, low, or very low, based on considerations of risk of bias, inconsistency, indirectness, imprecision, and publication bias.

## Results

### Study Selection

**Figure 1:**
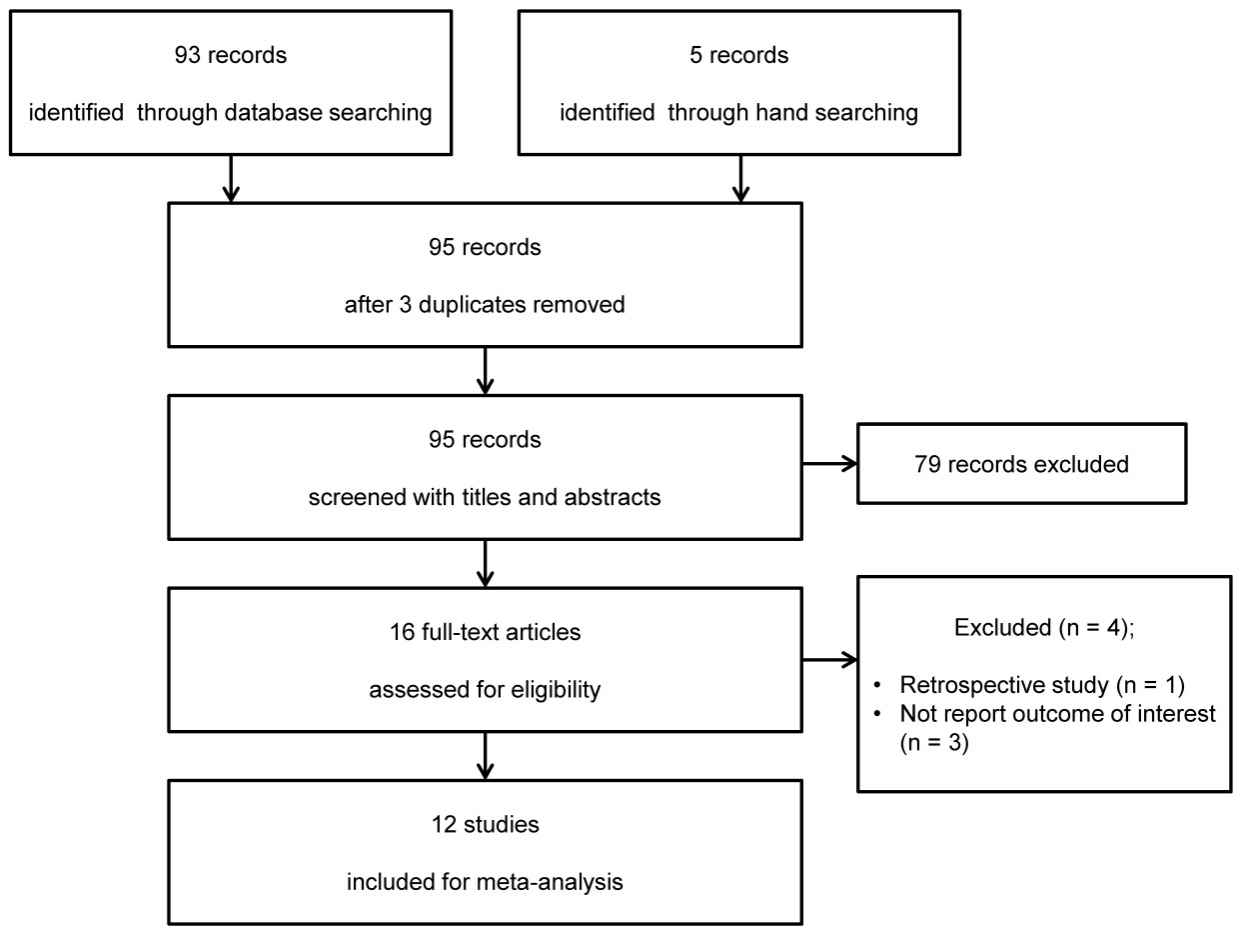
PRISMA flowchart of trial inclusion and exclusion.

From the MEDLINE, Embase, CENTRAL, and Google Scholar database search, 93 studies were initially evaluated, and a subsequent manual search revealed five additional studies. After adjusting for duplicates, 95 studies remained. Of these, 79 were excluded after reviewing the titles and abstracts. The remaining 16 studies were reviewed in detail, after which four studies were excluded because of their retrospective nature 19 or because they did not report any outcome of interest 20-22. Thus, 12 studies 7, 23-33 were eventually included in this systematic review and meta-analysis (Figure 1). The kappa value for selecting articles between the two reviewers was 0.826.

### Study Characteristics

The characteristics of the 12 RCTs that met the inclusion criteria are summarised in Table 1. Studies were conducted in the USA 23, 24, South Africa 25-27, Republic of Korea 7, 28, 29, India 30, 31, and Iran 32, 33.

The pharmacological strategies applied to prevent haemodynamic changes after intubation in parturient women with hypertensive disorders of pregnancy included the administration of lidocaine 25, magnesium sulphate 25, 26, esmolol 30, labetalol 24, nitroglycerin 23, 33, nifedipine 31, 33, hydralazine 33, fentanyl 27, 32, alfentanil 25, 27, remifentanil 28, 29, 32, 34, combination of magnesium sulphate and alfentanil 26, and combination of esmolol and lidocaine 30 (Table 1).

### Study Quality Assessment

The ROB assessment performed with the Cochrane tool for the included studies is presented in Table 2.

### Result Presentation

**Figure 2:**
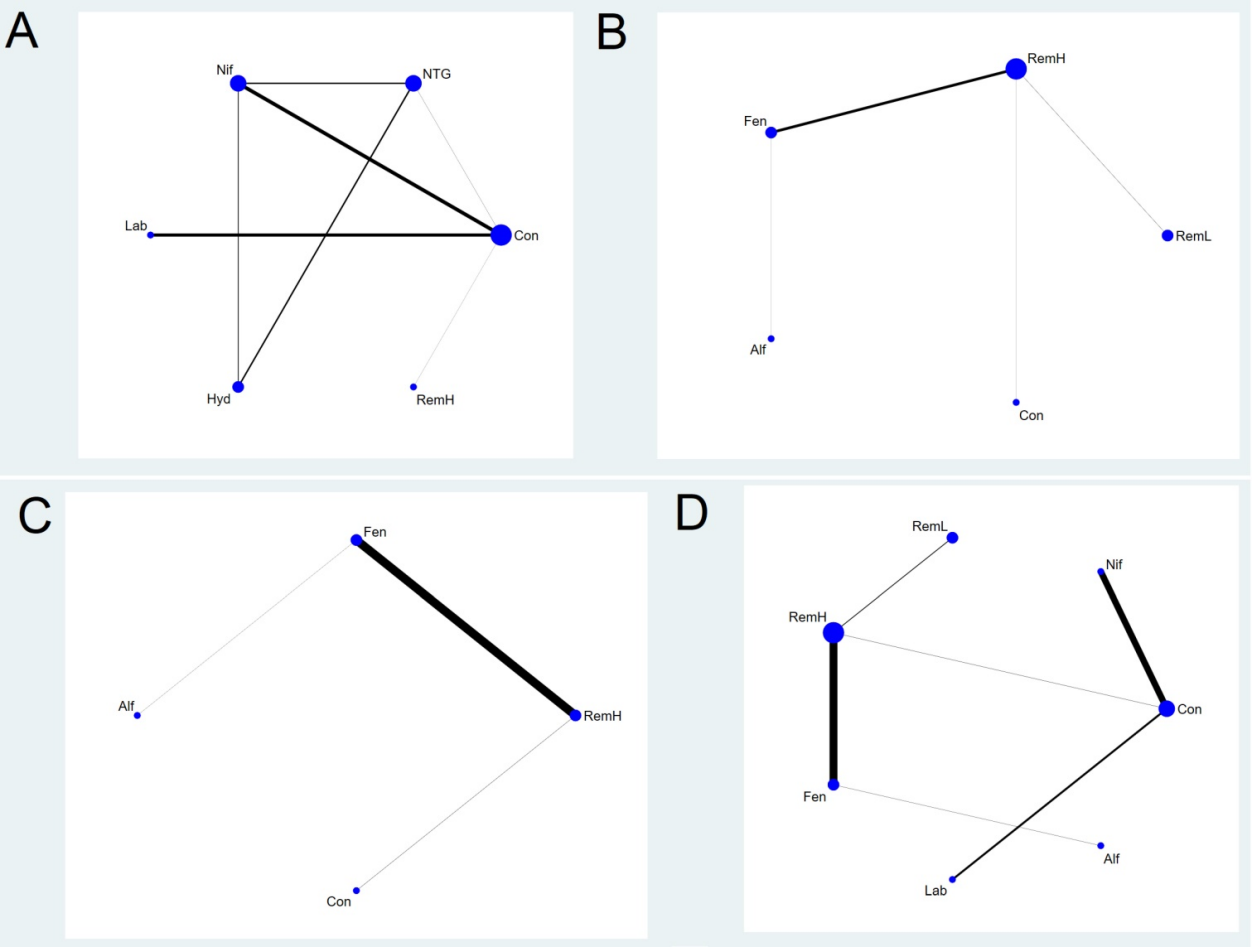
** Network plot of the included studies comparing different pharmacological strategies.** The nodes represent the pharmacological regimens used to prevent haemodynamic changes after intubation in parturient women with hypertensive disorders of pregnancy, and the edges show the available direct comparisons among them. Nodes and edges are weighted on the basis of the number of patients included and the inverse standard error of the effect. A) maximal mean arterial pressure, B) maximal systolic arterial pressure, C) maximal diastolic arterial pressure, D) maximal heart rate. Alf, alfentanil; Con, control; Fen, fentanyl; Hyd, hydralazine; Lab, labetalol; Nif, nifedipine; NTG, nitroglycerin; RemH, high dose of remifentanil; RemL, low dose of remifentanil.

**Figure 3:**
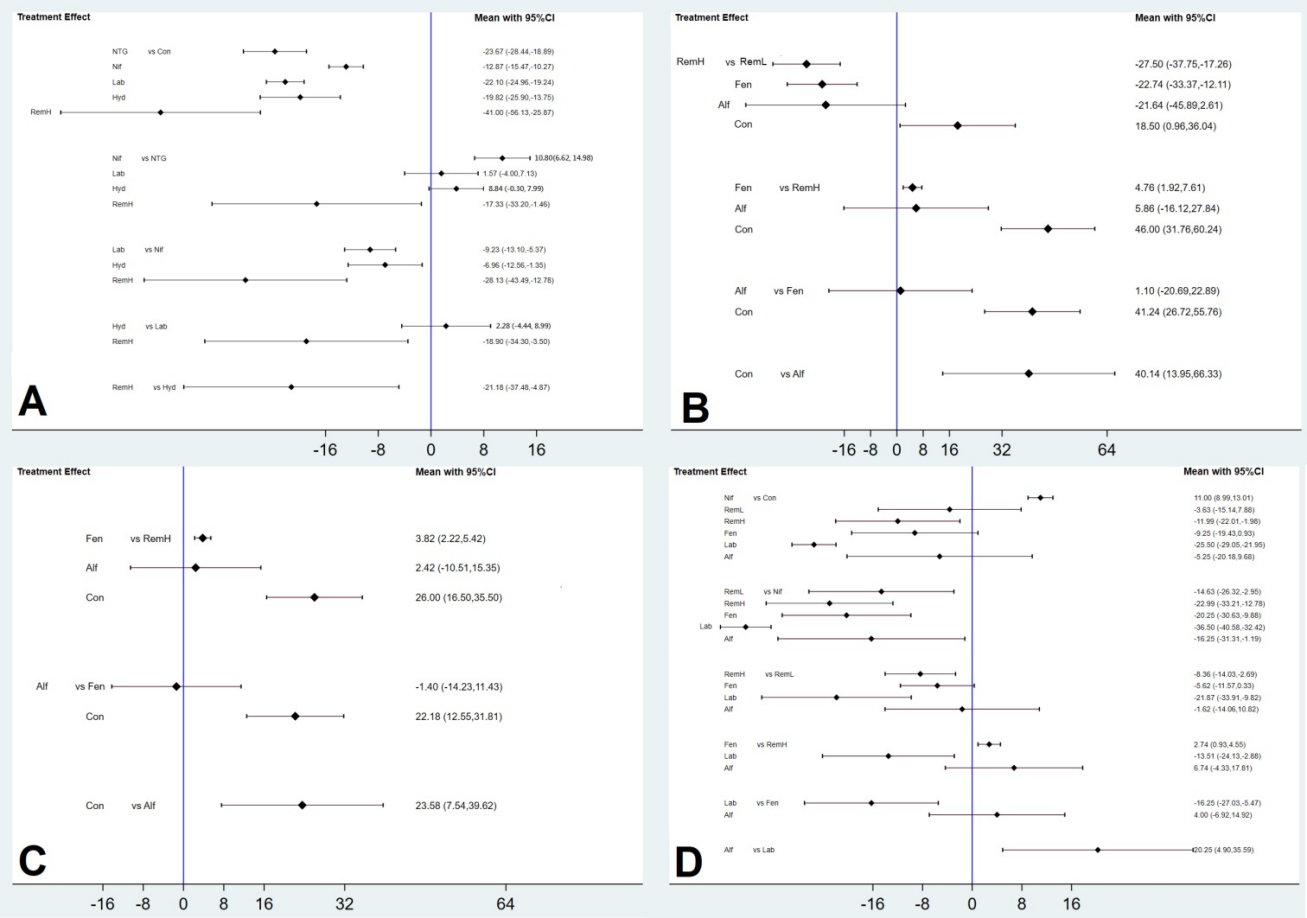
** Confidence interval plots between each management modality and the placebo group.** The diamond shape represents the mean summary effects; the black line, 95% CI. A) maximal mean arterial pressure, B) maximal systolic arterial pressure, C) maximal diastolic arterial pressure, D) maximal heart rate. CI, confidence interval; Alf, alfentanil; Con, control; Fen, fentanyl; Hyd, hydralazine; Lab, labetalol; Nif, nifedipine; NTG, nitroglycerin; RemH, high dose of remifentanil; RemL, low dose of remifentanil.

**Figure 4:**
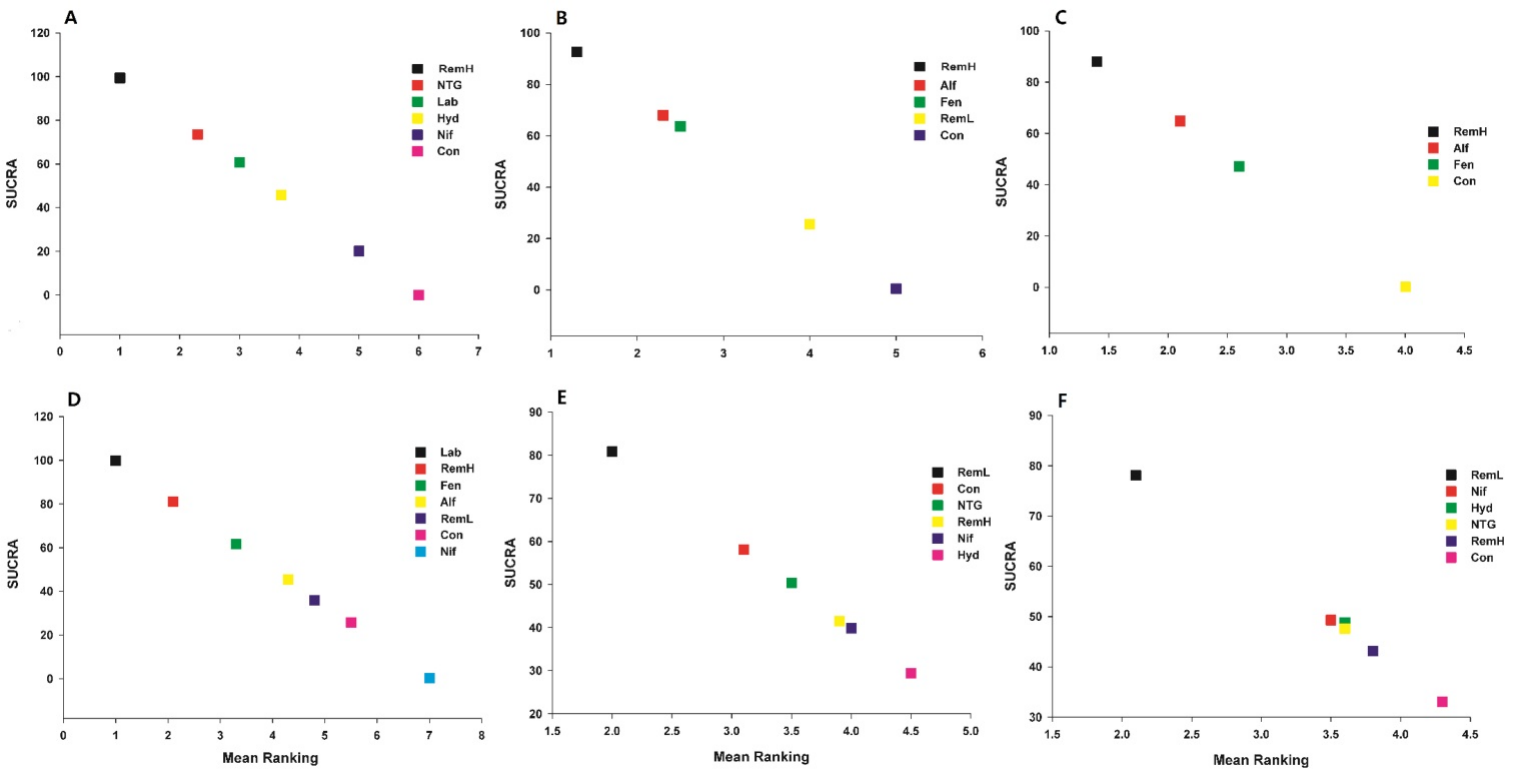
** Expected mean ranking and surface under the cumulative ranking curve (SUCRA) values.** The X-axis represents the expected mean ranking based on the SUCRA value, and the Y-axis represents the SUCRA value. A) maximal mean arterial pressure, B) maximal systolic arterial pressure, C) maximal diastolic arterial pressure, D) maximal heart rate, E) Apgar score at 1 min, and F) Apgar score at 5 min. Alf, alfentanil; Con, control; Fen, fentanyl; Hyd, hydralazine; Lab, labetalol; Nif, nifedipine; NTG, nitroglycerin; RemH, high dose of remifentanil; RemL, low dose of remifentanil.

For all outcomes, we presented the network plot (Figure 2), confidence interval plot (Figure 3), and expected mean ranking and SUCRA values for each pharmacological agent (Figure 4). Contribution plots, inconsistency plots between direct and indirect effect estimates for the same comparison, rankograms, and cumulative ranking curves are shown in Supplemental Figures S1, S2, S3, and S4, respectively. Apgar scores at 1 and 5 min in the network plot and confidence interval plot are shown in Supplemental Figures S5 and S6. Additionally, league tables of estimated effects of pharmacological strategies in network meta-analysis are shown in Supplemental Figure S7.

### Maximal Mean Arterial Pressure

Six pharmacological agents were compared in five studies (231 patients) for MMAP (Figure 2A) 23, 24, 31, 33, 34. Seven comparisons were performed using mixed evidence (both direct and indirect evidence) and eight using indirect evidence alone (Supplementary Figure S1).

There were two closed loops in the network related to the comparison of the MMAP, but one loop (nitroglycerin-nifedipine-hydralazine) was formed only in a multi-arm trial 33. There was no significant local inconsistency between direct and indirect point estimates (Supplementary Figure S2).

High doses of remifentanil (RemH) showed lower MMAP than those for hydralazine, labetalol, nifedipine, nitroglycerin and control; hydralazine showed lower MMAP than that for nifedipine and control; labetalol showed lower MMAP than that for nifedipine and control; nitroglycerin showed lower MMAP than those for nifedipine and control; nifedipine showed lower MMAP than that for control in terms of their 95% CIs (Figure 3A). The rankogram and cumulative ranking plot showed that RemH had the lowest MMAP (Figures S3A and S4A). The expected mean rankings and SUCRA values for each pharmacological agent in Figure 4A were highest for RemH (99.4%), followed by those for nitroglycerin (73.6%), labetalol (60.9%) and hydralazine (45.8%).

### Maximal Systolic Arterial Pressure

A total of eight studies (481 patients) considered MSAP. We excluded three studies from the NMA that were separated from the loops 26, 30, 33. Thus, a total of five studies (243 patients) were analysed 27-29, 32, 34. The network plot of all eligible comparisons for this endpoint is depicted in Figure 2B. Four comparisons were performed using mixed evidence (both direct and indirect evidence) and six using indirect evidence alone (Supplementary Figure S1B). As there was no closed loop in the network, inconsistency was not evaluated.

RemH showed lower MSAP than those for RemL, fentanyl, and control; fentanyl showed lower MSAP than those for RemL and control; alfentanil showed lower MSAP than that for control, and RemL showed lower MSAP than that for control (Figure 3B).

The rankogram and cumulative ranking plot showed that RemH had the lowest MSAP (Figures S3B and S4B). The expected mean rankings and SUCRA values for each pharmacological agent (Figure 4B) were highest for RemH (92.6%), followed by those for alfentanil (67.9%) and fentanyl (63.6%).

### Maximal Diastolic Arterial Pressure

A total of four studies (240 patients) measured MDAP. We excluded one study from the NMA because it was separated from the loops 33. Thus, a total of three studies (120 patients) were analysed 27, 32, 34. The network plot of all eligible comparisons for this endpoint is depicted in Figure 2C. Three comparisons were performed using mixed evidence (both direct and indirect evidence) and three using indirect evidence alone (Supplementary Figure S1C). As there was no closed loop in the network, inconsistency was not evaluated.

RemH showed lower MDAP than that for fentanyl and control; fentanyl showed lower MDAP than that for control; alfentanil showed lower MDAP than that for control (Figure 3C).

The rankogram and cumulative ranking plot showed that RemH had the lowest MDAP (Figures S3C and S4C). The expected mean rankings and SUCRA values for each pharmacological agent (Figure 4C) were highest for RemH (88.0%), followed by those for alfentanil (64.8%) and fentanyl (47.2%).

### Maximal Heart Rate

A total of 10 studies (531 patients) measured MHR. We excluded three studies from the NMA because they were separated from the loops 26, 30, 33. Thus, a total of seven studies (293 patients) were analysed 24, 27-29, 31, 32, 34. The network plot of all eligible comparisons for this endpoint is depicted in Figure 2D. Six comparisons were performed using mixed evidence (both direct and indirect evidence) and 15 using indirect evidence alone (Supplementary Figure S1D). As there was no closed loop in the network, inconsistency was not evaluated.

Labetalol showed lower MHR than that for RemH, fentanyl, alfentanil, RemL, control, and nifedipine. RemH showed lower MHR than that for fentanyl, RemL, control, and nifedipine. Fentanyl showed lower MHR than that for nifedipine. Alfentanil showed lower MHR than that for nifedipine. Nifedipine showed higher MHR than that for control (Figure 3D).

The rankogram and cumulative ranking plot showed that labetalol had the lowest MHR (Figure S3D and S4D). The expected mean rankings and SUCRA values for each pharmacological agent (Figure 4D) were highest for labetalol (99.9%), followed by those for RemH (81.2%) and fentanyl (61.6%).

### Apgar Score at 1 Minute

A total of nine studies (468 patients) measured the Apgar score at 1 min. Of those, seven reported the Apgar score at 1 min using a categorical variable (Apgar ≥ 7 vs. Apgar < 7) 23, 27-30, 33, 34, while four studies reported it using a continuous variable 26, 28, 29, 31; two studies reported both 28, 29. Thus, we analysed the Apgar score at 1 min using continuous or categorical variables separately.

Considering the four studies (191 patients) that measured the Apgar score at 1 min reporting continuous variables, we performed pair-wise meta-analysis because two studies were separated from the loops 26, 31 and the remaining two compared two identical groups (RemL vs. RemH) 28, 29. The Apgar score at 1 min was higher for RemL than for RemH (MD, 0.726; 95% CI, 0.056 to 1.396; I^2^ = 0.0%).

Among the seven studies (281 patients) that reported the Apgar score at 1 min as a categorical variable, two were excluded from the NMA because they were separated from the loops 27, 30. Thus, a total of five studies (293 patients) were analysed 23, 28, 29, 33, 34. The network plot of all eligible comparisons for this endpoint is depicted in Figure S5A.

One comparison (RemL vs. RemH) was conducted using direct evidence alone. Five comparisons were conducted using mixed evidence (both direct and indirect evidence) and nine using indirect evidence alone (Supplementary Figure S1E). As a closed loop (nitroglycerin-nifedipine-hydralazine) was formed in the multi-arm trial 33, inconsistency was not evaluated. RemH showed lower Apgar score at 1 min than RemL (Figure S6A).

The rankogram and cumulative ranking plot showed that RemL had the lowest incidence of lower Apgar score (Apgar < 7) (Figures S3E and S4E). The expected mean rankings and SUCRA values of each pharmacological agent in Figure 4E were highest for RemL (80.9%), followed by those for control (58.1%) and nitroglycerin (50.4%).

### Apgar Score at 5 minutes

A total of nine studies (468 patients) measured the Apgar score at 5 min. Of those, seven studies reported the Apgar score at 5 min as a categorical variable (Apgar ≥ 7 vs. Apgar < 7)^23^, ^27^-^30^, ^33^, ^34^, four reported it as a continuous variable^26^, ^28^, ^29^, ^31^, and two reported both^28^, ^29^. Thus, we analysed the Apgar score at 5 min using continuous or categorical variables separately.

Considering the four studies (191 patients) that reported the Apgar score at 5 min as a continuous variable, we performed pair-wise meta-analysis because two studies were separated from the loops^26^, ^31^ and the remaining two compared two identical groups (RemL vs. RemH)^28^, ^29^. There was no evidence of differences between the groups (MD, 0.359; 95% CI, -0.001 to 0.720; I^2^ = 0.0%).

Among the seven studies (281 patients) that reported the Apgar score at 5 min as a categorical variable, two that were separated from the loops were excluded from the NMA 27, 30. Thus, a total of five studies (293 patients) were analysed 23, 28, 29, 33, 34. The network plot of all eligible comparisons for this endpoint is depicted in Figure S5B.

One comparison (RemL vs. RemH) was conducted using direct evidence alone. Five comparisons were conducted using mixed evidence (both direct and indirect evidence) and nine using indirect evidence alone (Supplementary Figure S1F). As one closed loop (nitroglycerin-nifedipine-hydralazine) was formed in the multi-arm trial 33, inconsistency was not evaluated. There was no evidence of differences between pharmacological agents (Figure S6B).

The rankogram and cumulative ranking plot showed that RemL had the lowest incidence of lower Apgar score (Apgar < 7; Figures 4F and S3F). The expected mean rankings and SUCRA values (Figure 4F) were highest for RemL (78.1%), followed by those for nifedipine (49.3%) and hydralazine (48.8%).

## Discussion

Haemodynamic management in parturient women with hypertensive disorders of pregnancy undergoing caesarean section under general anaesthesia requires meticulous effort. These patients are more susceptible to hypertension and tachycardia after intubation, and physicians have adopted and compared several pharmacological strategies to avoid adverse outcomes such as intracranial pressure, cerebral haemorrhage, cardiac failure, and pulmonary oedema. To identify the most effective pharmacological strategy, we performed an NMA with 12 studies that compared arterial blood pressure, heart rate, and foetal outcomes.

Systolic, diastolic, and mean arterial blood pressures were compared between seven different pharmacological strategies—high and low doses of remifenatnil, alfentanil, fentanyl, nitroglycerin, hydralazine, labetalol, and nifedipine. Among these drugs, high dose of remifentanil produced the smallest changes in systolic, diastolic, and mean blood pressures and was therefore found to be the most effective drug in maintaining stable haemodynamic condition after intubation. Remifentanil's unique pharmacokinetic characteristics, due to its ultrashort effect and half-life, provided optimal analgesia and antihypertensive effects in women with hypertensive disorders of pregnancy. In addition, high dose of remifentanil (1.0 mcg/kg, 1.25 mcg/kg, and continuous infusion at a rate of 0.5 mcg/kg/min, 3 min prior to intubation) was significantly more effective than low dose of remifentanil (0.25 mcg/kg, 0.5 mcg/kg, and 0.75 mcg/kg) in controlling systolic arterial pressure. However, since delayed respiratory depression may occur in some newborns whose mothers are administered high dose of remifentanil, cautious dosing of this drug must be considered 35. In addition, high dose of remifentanil may result in hypotension in parturient women, leading to decreased uterine blood flow. However, the differences in Apgar scores extracted from the NMA were not significant between the various doses of remifentanil, and the data were insufficient to analyse hypotension events among different pharmacological strategies.

In addition to arterial blood pressure, the heart rates of parturient women after intubation were compared among five pharmacological strategies—high and low doses of remifentanil, labetalol, fentanyl, alfentanil, and nifedipine. In contrast with the results obtained for arterial blood pressure, labetalol was significantly most effective in maintaining baseline heart rate, followed by high doses of remifentanil and fentanyl. The α and β antagonistic effects of labetalol decrease both heart rate and blood pressure, and therefore, labetalol was more effective in controlling heart rate than other antihypertensive drugs. Tachycardia during induction may result in increased myocardial oxygen demand, and could be a risk factor for the development of myocardial ischaemia and infarction for patients with cardiovascular disease 36. Hence, labetalol could be effective for the management of heart rate in high-risk hypertensive pregnant women.

The main concern regarding the use of opioids and antihypertensive drugs is their effects on newborns. When the Apgar scores as categorical variables at 1 and 5 min were compared between drugs, there was no evidence of differences, except that high dose of remifentanil resulted in significantly lower Apgar scores than low dose of remifentanil. Moreover, pair-wise meta-analysis comparing the Apgar scores as continuous variables at 1 and 5 min between low-dose and high-dose remifentanil showed no evidence of difference between the two groups. Although opioid use prior to delivery of the foetus is still controversial issue, we should be flexible on the change in favour of opioids administration which can be accompanied with greater benefits than not using them for both the mother and the foetus. In pregnant women with serious condition which is mandatory to maintain adequate haemodynamic state, the remifentanil can be a main option.

Although there was no evidence of significant differences in almost all comparisons and no cases of prolonged neurological adverse effects in the included studies, some studies reported respiratory depression, and sometimes tracheal intubation was required even in elective, uncomplicated term pregnancies 35, 37. As the birth weight of newborns decreases 38 and the incidence of respiratory distress syndrome increases in infants of mothers with hypertensive disorders of pregnancy 39, meticulous attention is required when applying pharmacological strategies to prevent haemodynamic changes after intubation in such mothers. Overall, the results of our NMA suggest that high dose of remifentanil reduces blood pressure during intubation in parturient women with hypertensive disorders of pregnancy.

There were several limitations in this study. First, as our NMA included 12 studies conducted in different clinical centres, methodological heterogeneity was present, and some study designs were not presented in sufficient detail. Additionally, some studies 26, 30, 33 were separated from the loops and could not be compared; hence, the data concerning magnesium sulphate, esmolol, and lidocaine were excluded from this NMA. Well-designed, large scale RCTs that include various drugs should be conducted in the future to complement our study findings.

In conclusion, hypertension and tachycardia during intubation for general anaesthesia in patients with hypertensive disorders of pregnancy could be lethal. Our study showed that remifentanil and labetalol were the most effective treatments for the management of blood pressure and heart rate, respectively. However, their effects in newborns should be considered and the appropriate dosage of these drugs should be investigated in the future.

## Supplementary Material

Supplementary figures.

## Figures and Tables

**Table 1:** Summary of randomised controlled trials included in the network meta-analysis

First author, year	Country	Medication	Dosage	No.	Endpoints
**Allen RW, 1991^25^**	South Africa	Lidocaine	1.5 mg/kg	21	SAP
		Alfentanil	10 mcg/kg	24	
		Mg sulphate	40 mg/kg	24	
**Ashton WB, 1991^26^**	South Africa	Mg sulphate	40 mg/kg	19	SAP
		Mg sulphate +alfentanil	30 mg/kg + 7.4 mcg/kg	19	
**Bansal S, 2005^30^**	India	Esmolol	1 mg/kg	20	SAP, HR, Apgar
		Esmolol	2 mg/kg	20	
		Esmolol + lidocaine (1)	1 mg/kg + 1.5 mg/kg	20	
		Esmolol + lidocaine (2)	2 mg/kg + 1.5 mg/kg	20	
**Hood D, 1985^23^**	United States	Control	N/D	10	MAP, HR, Apgar
		Nitroglycerin	200 mcg/ml until BP lowered 20%	9	
**Kumar N, 1993^31^**	India	Control	Capsule (similar physical characteristic)	15	HR, MAP, Apgar
		Nifedipine	10 mg PO (20 min before induction)	15	
**Park BY, 2011^28^**	South Korea	Remifentanil (1)	0.5 mcg/kg	24	SAP, HR, Apgar
		Remifentanil (2)	1 mcg/kg	24	
**Pournajafian A, 2012^32^**	Iran	Remifentanil	0.5 mcg/kg/min	20	SAP, DAP, HR
		Fentanyl	50 mcg	18	
**Ramanathan J, 1988^24^**	United States	Control	N/D	10	MAP, HR, Apgar R
		Labetalol	20 mg→10 mg increment every 2 min	10	
**Rout CC, 1990^27^**	South Africa	Fentanyl	2.5 mcg/kg	20	HR, SAP, DAP, Apgar
		Alfentanil	10 mcg/kg	20	
**Safavi M, 2011^33^**	Iran	Hydralazine	5-10 mg IV	40	SAP, MAP, DAP, HR, Apgar
		Nitroglycerin	5 mcg/min IV continuous	40	
		Nifedipine	10 mg sublingual	40	
**Yoo KY, 2009^34^**	South Korea	Control	N/D	21	SAP, MAP, DAP, HR, Apgar
		Remifentanil	1.0 mcg/kg	21	
**Yoo KY, 2013^29^**	South Korea	Remifentanil (1)	0.25 mcg/kg	15	SAP, HR, BIS, Apgar
		Remifentanil (2)	0.5 mcg/kg	15	
		Remifentanil (3)	0.75 mcg/kg	15	
		Remifentanil (4)	1.0 mcg/kg	15	
		Remifentanil (5)	1.25 mcg/kg	15	

No., number of patients; Mg, magnesium; SAP, systolic arterial pressure; MAP, mean arterial pressure; DAP, diastolic arterial pressure; HR, heart rate; Apgar, Apgar score; BIS, bispectral index; N/D = not defined.

**Table 2:** Risk of bias

First author, year	Bias arising from the randomisation process	Bias due to deviations from intended intervention	Bias due to missing outcome data	Bias in measurement of the outcome	Bias in selection of the reported results	Overall Bias
Allen RW, 1991^25^	Some concerns	Low risk	Low risk	Low risk	Low risk	Some concerns
Ashton WB, 1991^26^	Some concerns	Low risk	Low risk	Low risk	Low risk	Some concerns
Bansal S, 2005^30^	Low risk	Low risk	Low risk	Low risk	Low risk	Low risk
Hood D, 1985^23^	Some concerns	Low risk	Low risk	Low risk	Low risk	Some concerns
Kumar N 1993^31^	Low risk	Low risk	Low risk	Low risk	Low risk	Low risk
Park BY 2011^28^	Low risk	Low risk	Low risk	Low risk	Low risk	Low risk
Pournajafian A 2012^32^	Some concerns	Low risk	Low risk	Low risk	Low risk	Some concerns
Ramanathan J 1988^24^	Some concerns	Low risk	Low risk	Low risk	Low risk	Some concerns
Rout CC 1990^27^	Some concerns	Low risk	Low risk	Low risk	Low risk	Some concerns
Safavi M 2011^33^	Low risk	Low risk	Low risk	Low risk	Low risk	Low risk
Yoo KY 2009^34^	Low risk	Low risk	Low risk	Low risk	Low risk	Low risk
Yoo KY 2013^29^	Low risk	Low risk	Low risk	Low risk	Low risk	Low risk

**Table 3:** Grading of Recommendations, Assessment, Development, and Evaluation (GRADE) evidence quality for each outcome

Outcomes	No. of studies	Quality assessment					Quality
		Risk of bias	Inconsistency	Indirectness	Imprecision	Publication bias	
Maximal MAP	5	not serious	not serious	not serious	serious	none	⨁⨁⨁◯Moderate
Maximal SAP	8	not serious	not serious	not serious	serious	none	⨁⨁⨁◯Moderate
Maximal DAP	4	not serious	not serious	not serious	serious	none	⨁⨁⨁◯Moderate
Maximal HR	10	not serious	not serious	not serious	serious	none	⨁⨁⨁◯Moderate
Apgar score at 1 min	9	not serious	not serious	not serious	serious	none	⨁⨁⨁◯Moderate
Apgar score at 5 min	9	not serious	not serious	not serious	serious	none	⨁⨁⨁◯Moderate

MAP, mean arterial pressure; SAP, systolic arterial pressure; DAP, diastolic arterial pressure; HR, heart rate
