# Supplementary material for: Pharmacological strategies to prevent haemodynamic changes after intubation in parturient women with hypertensive disorders of pregnancy: A network meta-analysis
Source: Int J Med Sci. 2021 Jan 1;18(4):1039–50. doi: 10.7150/ijms.54002 (PMC7807187; doi:10.7150/ijms.54002)
Supplement: Supplementary file 1 — Supplementary figures. [file ijmsv18p1039s1.pdf]

Supplementary figure S1. Contribution plots. Rows correspond to mixed and indirect evidence, and columns correspond to direct evidence. The percentage contribution of each direct comparison to the network summary is presented in the entire network row. The sizes of the boxes are proportional to the percentage contribution of each direct estimate to the network meta-analysis estimates and to the entire network. The last row shows the number of included direct comparisons.

A) maximal mean arterial pressure, B) maximal systolic arterial pressure, C) maximal diastolic arterial pressure, D) maximal heart rate, E) Apgar score at 1 min and F) APGAR score at 5 min.

Supplementary figure S2. Inconsistency plots between direct and indirect effect estimates for the same comparison. Inconsistency factor (IF) as the absolute difference with 95% confidence interval (CI) between direct and indirect estimates for each paired comparison. IF values close to 0 indicate that the two sources are in agreement. A) maximal mean arterial pressure, 1: control, 2: nitroglycerin, 3: nifedipine

Supplementary figure S3. The profiles indicate the probabilities for each treatment to assume any of the possible ranks, i.e. the probability that a given treatment ranks first, second, third, etc., among all treatments evaluated in the network meta-analysis.

A) maximal mean arterial pressure, B) maximal systolic arterial pressure, C) maximal diastolic arterial pressure, D) maximal heart rate, E) Apgar score at 1 min, and F) Apgar score at 5 min.

Alf, alfentanil; Con, control; Fen, fentanyl; Hyd, hydralazine; Lab, labetalol; Nif, nifedipine; NTG, nitroglycerin; RemH, high dose of remifentanil; RemL, low dose of remifentanil.

Supplementary figure S4. Cumulative ranking curve plots. The profile indicates the sum of the probabilities from those ranked first, second, third, etc. The closer the cumulative ranking curve (SUCRA) value is to its maximum value of 100% the higher the treatment is ranked relative to all other treatments.

A) maximal mean arterial pressure, B) maximal systolic arterial pressure, C) maximal diastolic arterial pressure, D) maximal heart rate, E) Apgar score at 1 min, and F) Apgar score at 5 min.

Alf, alfentanil; Con, control; Fen, fentanyl; Hyd, hydralazine; Lab, labetalol; Nif, nifedipine; NTG, nitroglycerin; RemH, high dose of remifentanil; RemL, low dose of remifentanil.

Supplementary figure S5. Network plots of the included studies comparing different pharmacological strategies. The nodes represent the pharmacological regimens used to prevent haemodynamic changes after intubation in parturient women with hypertensive disorders of pregnancy, and the edges show the available direct comparisons among them. Nodes and edges are weighted on the basis of the number of patients included and the inverse standard error of the effect. A) Apgar score at 1 min; and B) Apgar score at 5 min.

Alf, alfentanil; Con, control; Fen, fentanyl; Hyd, hydralazine; Lab, labetalol; Nif, nifedipine; NTG, nitroglycerin; RemH, high dose of remifentanil; RemL, low dose of remifentanil.

Supplementary figure S6. Confidence interval plots between each management modality and the placebo group. The diamond shape represents the mean summary effects; the black line, 95% CI. A) Apgar score at 1 min, and B) Apgar score at 5 min.

CI, confidence interval; Alf, alfentanil; Con, control; Fen, fentanyl; Hyd, hydralazine; Lab,

labetalol; Nif, nifedipine; NTG, nitroglycerin; RemH, high dose of remifentanil; RemL, low dose of remifentanil.

Supplementary figure S7. League tables of estimated effects of pharmacological agents in network meta-analysis. Estimated effects are presented as the mean summary effects relative with the 95% CI. A) maximal mean arterial pressure, B) maximal systolic arterial pressure, C) maximal diastolic arterial pressure, D) maximal heart rate, E) Apgar score at 1 min, and F) Apgar score at 5 min.

Alf, alfentanil; Con, control; Fen, fentanyl; Hyd, hydralazine; Lab, labetalol; Nif, nifedipine; NTG, nitroglycerin; RemH, high dose of remifentanil; RemL, low dose of remifentanil.

Supplementary figure S1. Contribution plots.

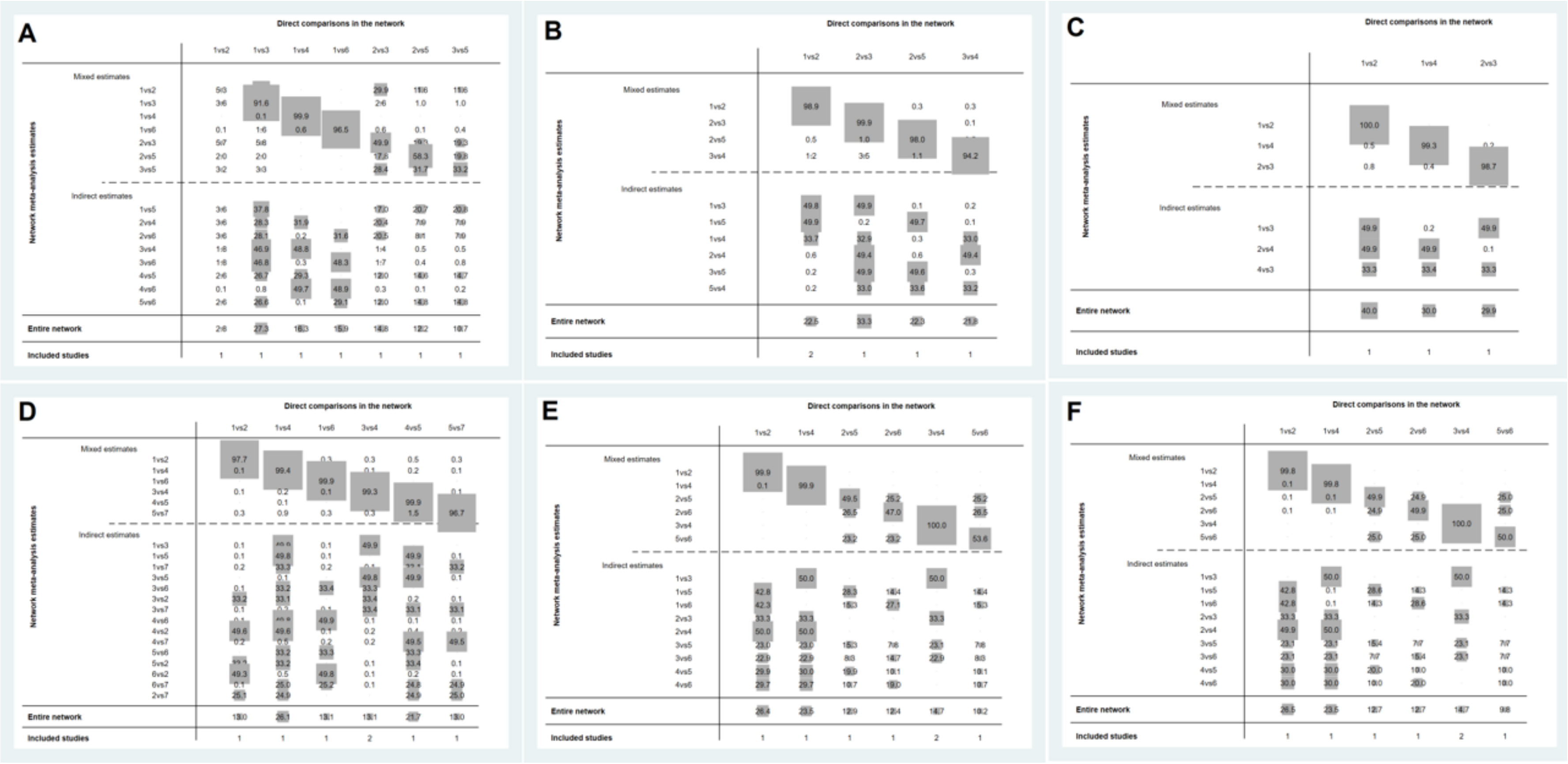

Supplementary figure S2. Rankograms

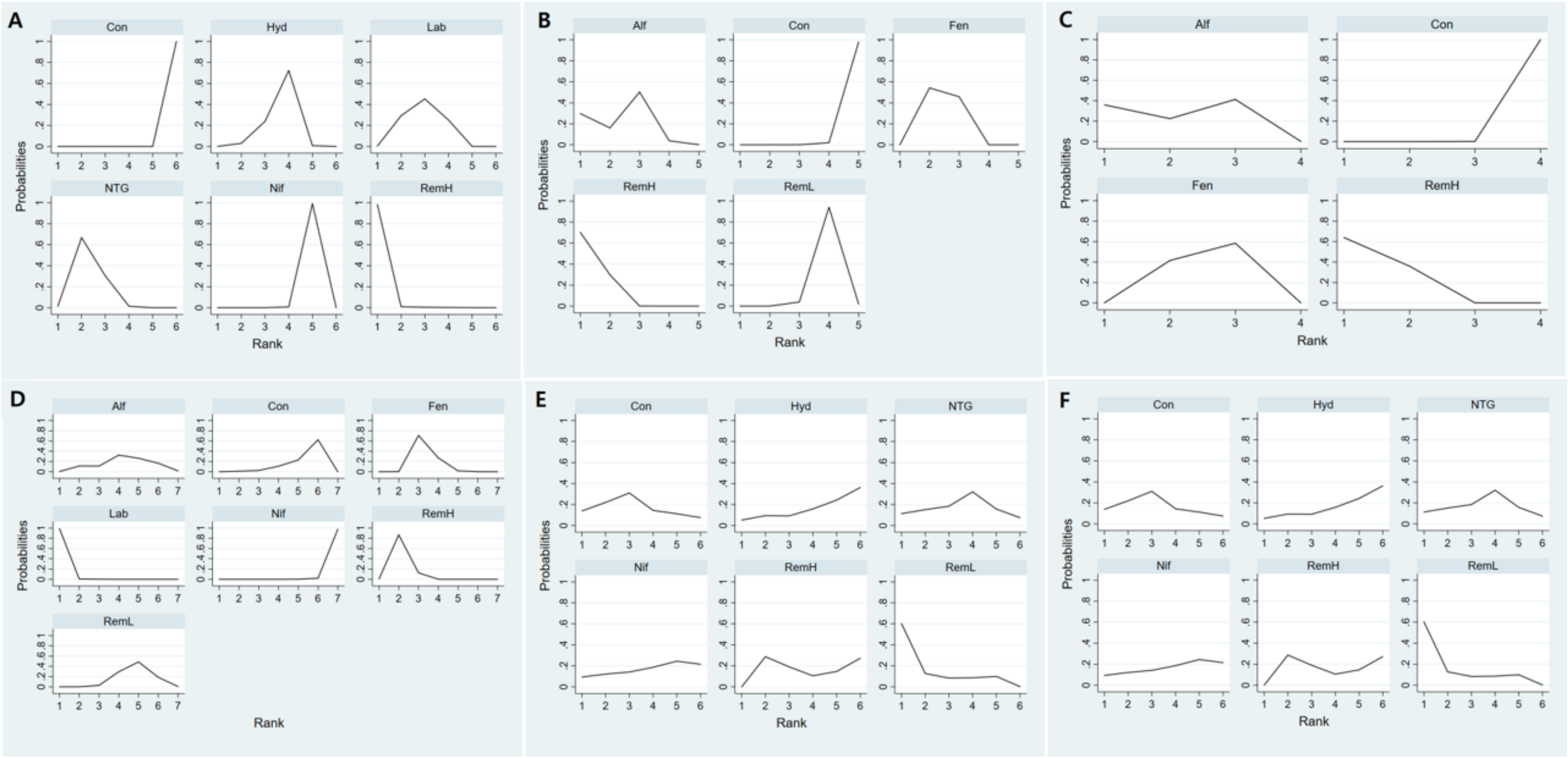

Supplementary figure S3. Inconsistency plots between direct and indirect effect estimates for the same comparison.

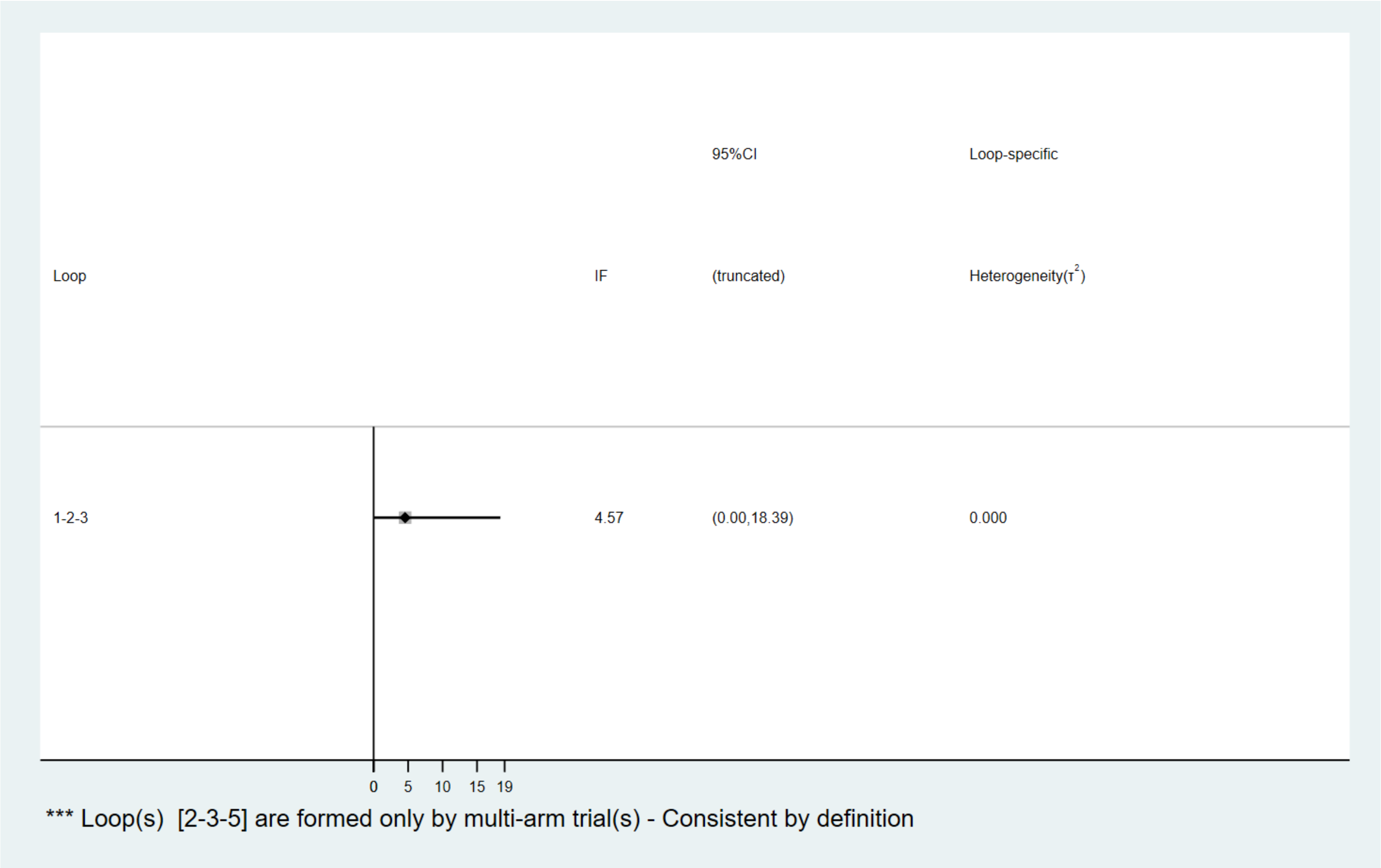

Supplementary figure S4. Cumulative ranking curve plots

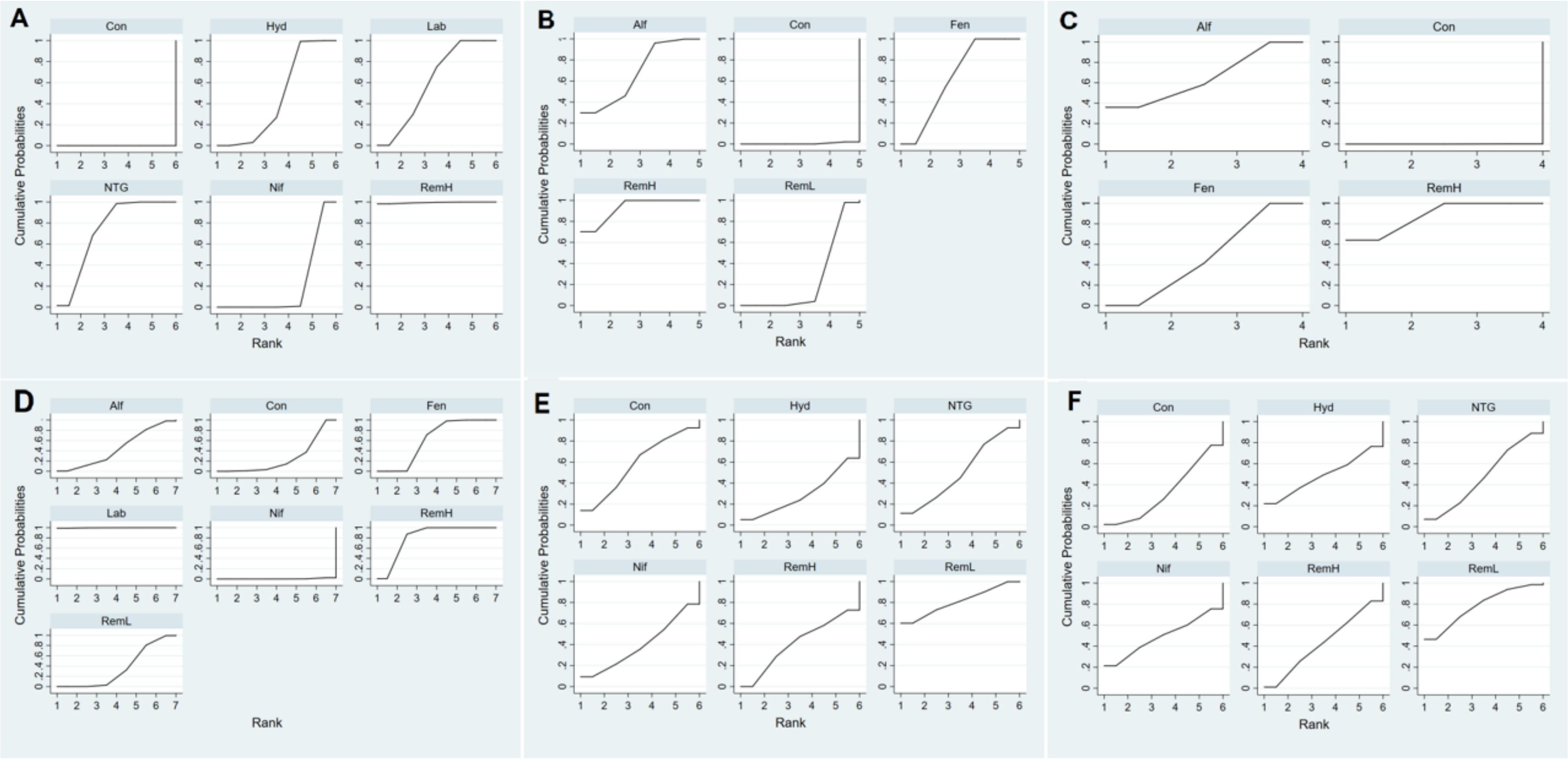

Supplementary figure S5. Network plots

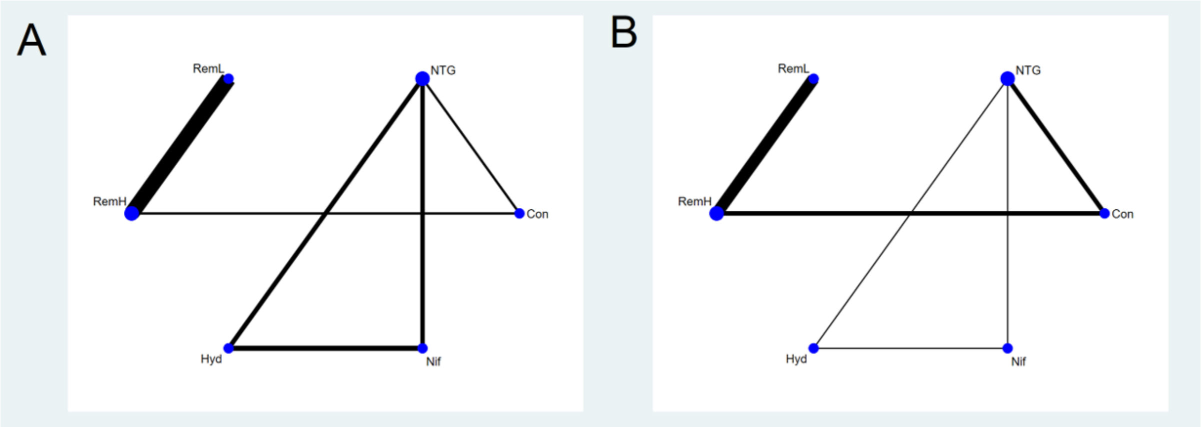

Supplementary figure S6. Confidence interval plots

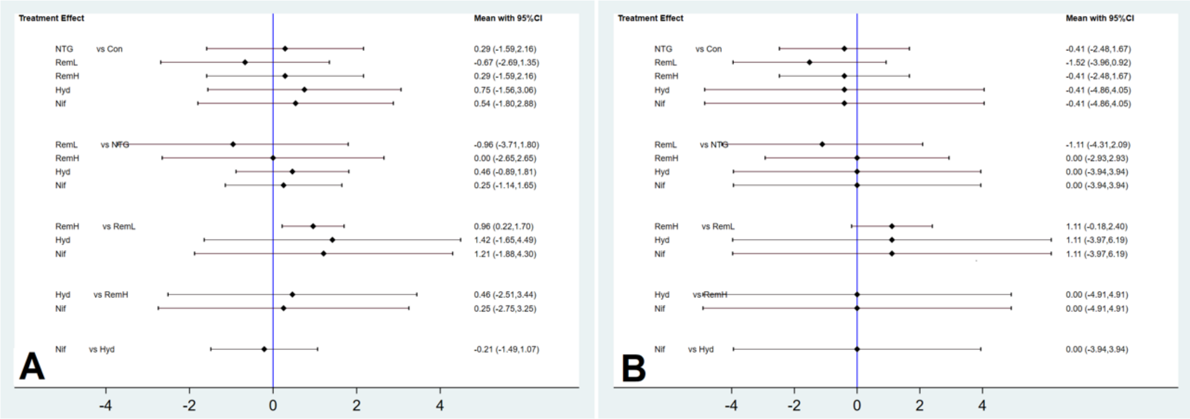

Supplementary figure S7. League tables

A

**RemH**	17.33 (1.46,33.20)	18.90 (3.50,34.30)	21.18 (4.87,37.48)	28.13 (12.78,43.49)	41.00 (25.87,56.13)
-17.33 (-33.20,-1.46)	**NTG**	1.57 (-4.00,7.13)	3.84 (-0.30,7.99)	10.80 (6.62,14.98)	23.67 (18.89,28.44)
-18.90 (-34.30,-3.50)	-1.57 (-7.13,4.00)	**Lab**	2.28 (-4.44,8.99)	9.23 (5.37,13.10)	22.10 (19.24,24.96)
-21.18 (-37.48,-4.87)	-3.84 (-7.99,0.30)	-2.28 (-8.99,4.44)	**Hyd**	6.96 (1.35,12.56)	19.82 (13.75,25.90)
-28.13 (-43.49,-12.78)	-10.80 (-14.98,-6.62)	-9.23 (-13.10,-5.37)	-6.96 (-12.56,-1.35)	**Nif**	12.87 (10.27,15.47)
-41.00 (-56.13,-25.87)	-23.67 (-28.44,-18.89)	-22.10 (-24.96,-19.24)	-19.82 (-25.90,-13.75)	-12.87 (-15.47,-10.27)	**Con**

B

**RemH**	5.86 (-16.12,27.84)	4.76 (1.92,7.61)	27.50 (17.26,37.75)	46.00 (31.76,60.24)
-5.86 (-27.84,16.12)	**Alf**	-1.10 (-22.89,20.69)	21.64 (-2.61,45.89)	40.14 (13.95,66.33)
-4.76 (-7.61,-1.92)	1.10 (-20.69,22.89)	**Fen**	22.74 (12.11,33.37)	41.24 (26.72,55.76)
-27.50 (-37.75,-17.26)	-21.64 (-45.89,2.61)	-22.74 (-33.37,-12.11)	**RemL**	18.50 (0.96,36.04)
-46.00 (-60.24,-31.76)	-40.14 (-66.33,-13.95)	-41.24 (-55.76,-26.72)	-18.50 (-36.04,-0.96)	**Con**

C

**RemH**	2.42 (10.51,15.35)	3.82 (2.22,5.42)	26.00 (16.50,35.50)
-2.42 (-15.35,10.51)	**Alf**	1.40 (-11.43,14.23)	23.58 (7.54,39.62)
-3.82 (-5.42,-2.22)	-1.40 (-14.23,11.43)	**Fen**	22.18 (12.55,31.81)
-26.00 (-35.50,-16.50)	-23.58 (-39.62,-7.54)	-22.18 (-31.81,-12.55)	**Con**

D

**Lab**	13.51 (2.88,24.13)	16.25 (5.47,27.03)	20.25 (4.90,35.59)	21.87 (9.82,33.91)	25.50 (21.95,29.05)	36.50 (32.42,40.58)
-13.51 (-24.13,-2.88)	**RemH**	2.74 (0.93,4.55)	6.74 (-4.33,17.81)	8.36 (2.69,14.03)	11.99 (1.98,22.01)	22.99 (12.78,33.21)
-16.25 (-27.03,-5.47)	-2.74 (-4.55,-0.93)	**Fen**	4.00 (-6.92,14.92)	5.62 (-0.33,11.57)	9.25 (-0.93,19.43)	20.25 (9.88,30.63)
-20.25 (-35.59,-4.90)	-6.74 (-17.81,4.33)	-4.00 (-14.92,6.92)	**Alf**	1.62 (-10.82,14.06)	5.25 (-9.68,20.18)	16.25 (1.19,31.31)
-21.87 (-33.91,-9.82)	-8.36 (-14.03,-2.69)	-5.62 (-11.57,0.33)	-1.62 (-14.06,10.82)	**RemL**	3.63 (-7.88,15.14)	14.63 (2.95,26.32)
-25.50 (-29.05,-21.95)	-11.99 (-22.01,-1.98)	-9.25 (-19.43,0.93)	-5.25 (-20.18,9.68)	-3.63 (-15.14,7.88)	**Con**	11.00 (8.99,13.01)
-36.50 (-40.58,-32.42)	-22.99 (-33.21,-12.78)	-20.25 (-30.63,-9.88)	-16.25 (-31.31,-1.19)	-14.63 (-26.32,-2.95)	-11.00 (-13.01,-8.99)	**Nif**

E

**RemL**	0.67 (1.35,2.69)	0.96 (-1.80,3.71)	0.96 (0.22,1.70)	1.21 (-1.88,4.30)	1.42 (-1.65,4.49)
-0.67 (-2.69,1.35)	**Con**	0.29 (-1.59,2.16)	0.29 (-1.59,2.16)	0.54 (-1.80,2.88)	0.75 (-1.56,3.06)
-0.96 (-3.71,1.80)	-0.29 (-2.16,1.59)	**NTG**	0.00 (-2.65,2.65)	0.25 (-1.14,1.65)	0.46 (-0.89,1.81)
-0.96 (-1.70,-0.22)	-0.29 (-2.16,1.59)	-0.00 (-2.65,2.65)	**RemH**	0.25 (-2.75,3.25)	0.46 (-2.51,3.44)
-1.21 (-4.30,1.88)	-0.54 (-2.88,1.80)	-0.25 (-1.65,1.14)	-0.25 (-3.25,2.75)	**Nif**	0.21 (-1.07,1.49)
-1.42 (-4.49,1.65)	-0.75 (-3.06,1.56)	-0.46 (-1.81,0.89)	-0.46 (-3.44,2.51)	-0.21 (-1.49,1.07)	**Hyd**

F

**RemL**	1.11 (-3.97,6.19)	1.11 (-3.97,6.19)	1.11 (-2.09,4.31)	1.11 (-0.18,2.40)	1.52 (-0.92,3.96)
-1.11 (-6.19,3.97)	**Nif**	0.00 (-3.94,3.94)	0.00 (-3.94,3.94)	0.00 (-4.91,4.91)	0.41 (-4.05,4.86)
-1.11 (-6.19,3.97)	-0.00 (-3.94,3.94)	**Hyd**	0.00 (-3.94,3.94)	0.00 (-4.91,4.91)	0.41 (-4.05,4.86)
-1.11 (-4.31,2.09)	-0.00 (-3.94,3.94)	-0.00 (-3.94,3.94)	**NTG**	0.00 (-2.93,2.93)	0.41 (-1.67,2.48)
-1.11 (-2.40,0.18)	-0.00 (-4.91,4.91)	-0.00 (-4.91,4.91)	-0.00 (-2.93,2.93)	**RemH**	0.41 (-1.67,2.48)
-1.52 (-3.96,0.92)	-0.41 (-4.86,4.05)	-0.41 (-4.86,4.05)	-0.41 (-2.48,1.67)	-0.41 (-2.48,1.67)	**Con**
